# Supplementary material for: An electron-blocking interface for garnet-based quasi-solid-state lithium-metal batteries to improve lifespan
Source: Nat Commun. 2024 Jun 22;15:5325. doi: 10.1038/s41467-024-49715-x (PMC11193789; doi:10.1038/s41467-024-49715-x)
Supplement: Supplementary file 1 — Supplementary Information [file 41467_2024_49715_MOESM1_ESM.pdf]

Supplementary Information for

An electron-blocking interface for garnet-based quasi-solid-state lithium-metal batteries to improve lifespan

Chang Zhang[1,2], Jiameng Yu[1], Yuanyuan Cui[3]*, Yinjie Lv[1], Yue Zhang[1], Tianyi Gao[1], Yuxi He[1], Xin Chen[1], Tao Li[4,5], Tianquan Lin[4,5], Qixi Mi[1], Yi Yu[1,2] and Wei Liu[1,2]*

[1]*School of Physical Science and Technology, ShanghaiTech University, Shanghai 201210, China*
[2]*Shanghai Key Laboratory of High-resolution Electron Microscopy, ShanghaiTech University; Shanghai, 201210, China*
[3]*School of Materials Science and Engineering, Shanghai University, Shanghai, 200444, China*
[4]*School of Materials Science and Engineering, Shanghai Jiao Tong University, Shanghai, 200240, China*
[5]*Zhangjiang Institute for Advanced Study (ZIAS), Shanghai Jiao Tong University, Shanghai 201210, China*

* Corresponding author. E-mail: cui-yy@shu.edu.cn, liuwei1@shanghaitech.edu.cn

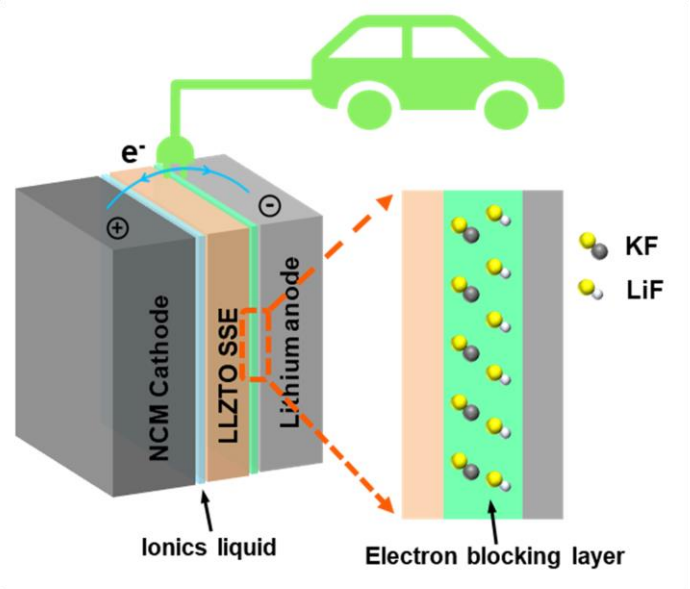

Figure S1. Schematic illustration of garnet-base Li-NCM solid-state lithium metal battery and enlarged image of interface between lithium metal and LLZTO solid-solid electrolyte.

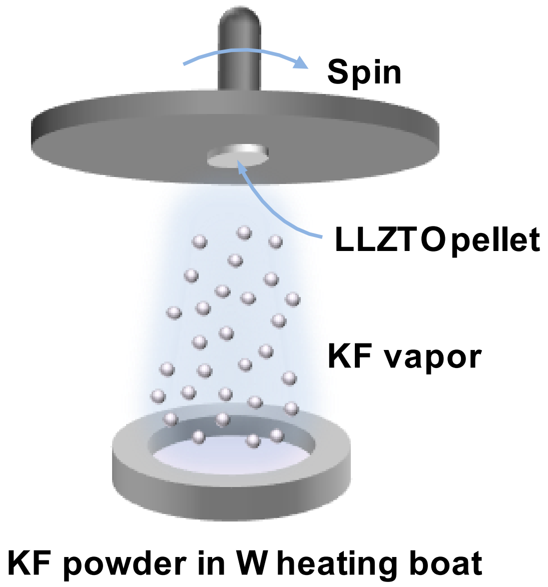

Figure S2. Schematic illustration of KF buffer layer deposition process by vacuum thermal evaporation.

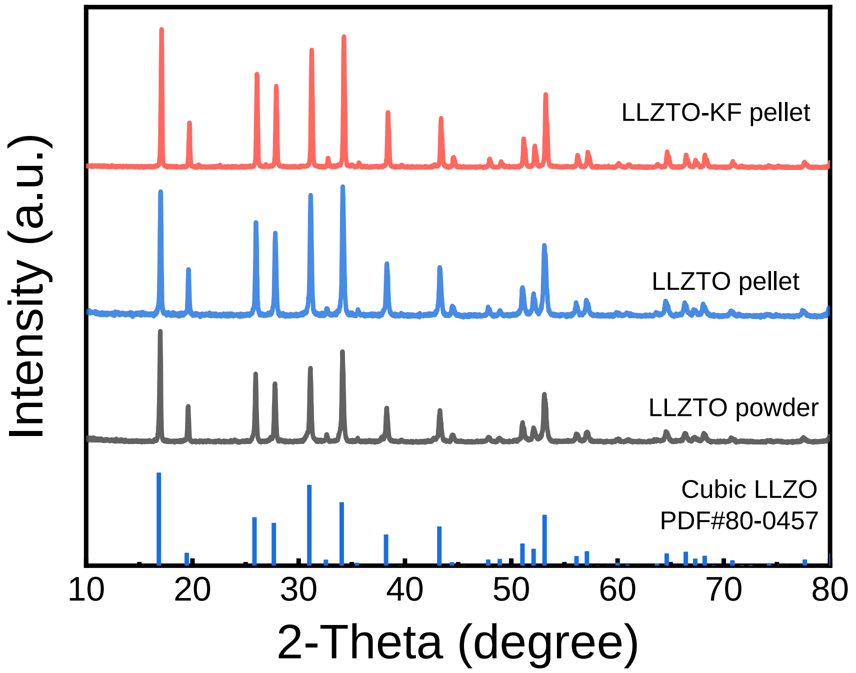

Figure S3. XRD patterns of (a) prepared LLZTO powder, LLZTO pellet and LLZTO-KF pellet.

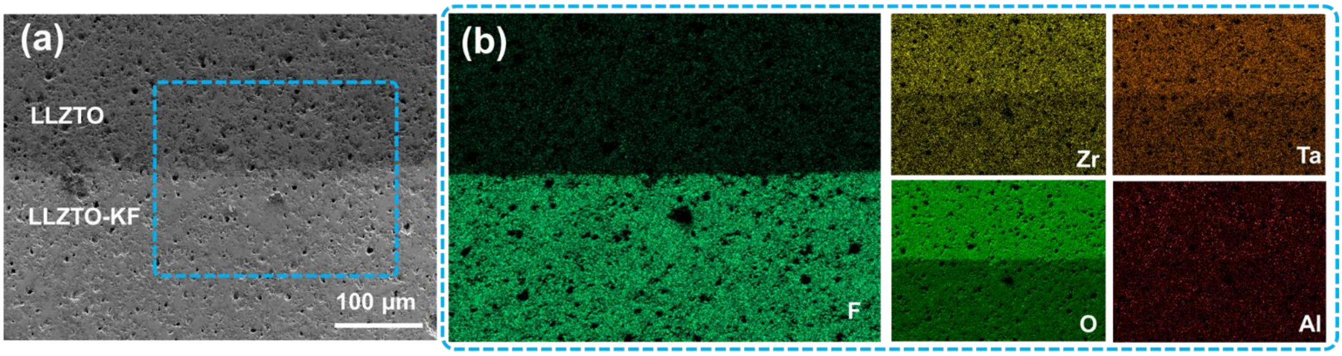

Figure S4. Morphology of prepared LLZTO pellet and LLZTO-KF pellet. (a), Surface contrast of SEM image of LLZTO pellet with/without KF layer. (b) Corresponding EDS mappings of F, Zr, Ta, O and Al elements.

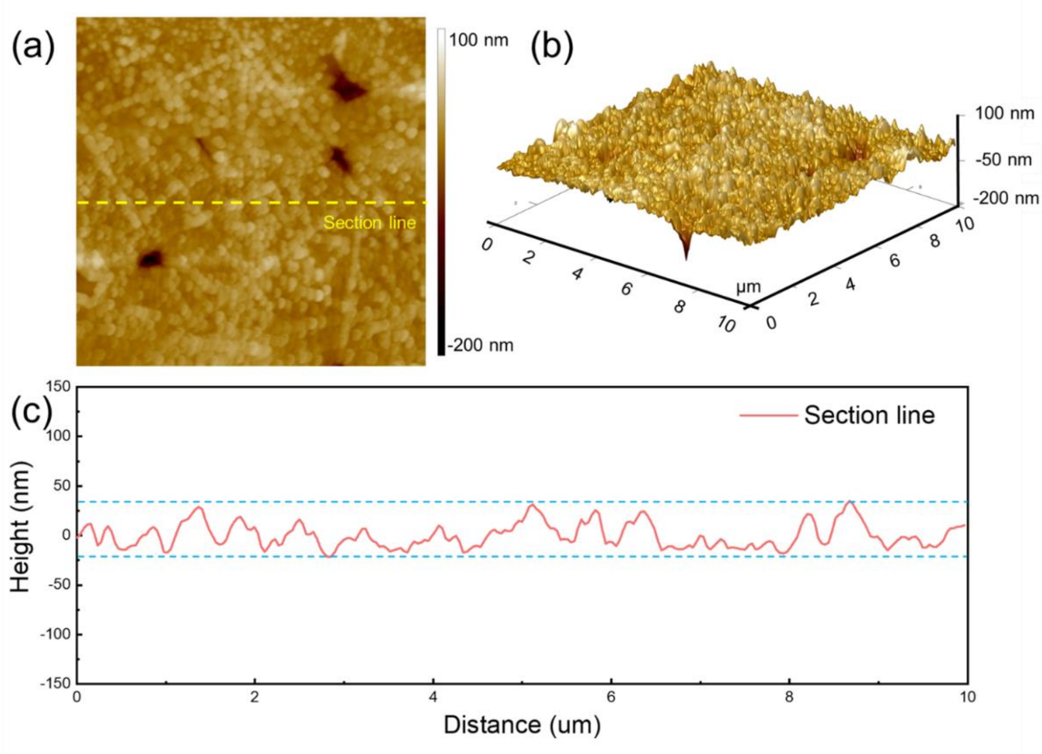

Figure S5. (a) AFM topography image of LLZTO-KF (10 × 10 μm). (b) 3D AFM topographic image of (a). (c) Height profiles along the yellow line in the 2D image of (a).

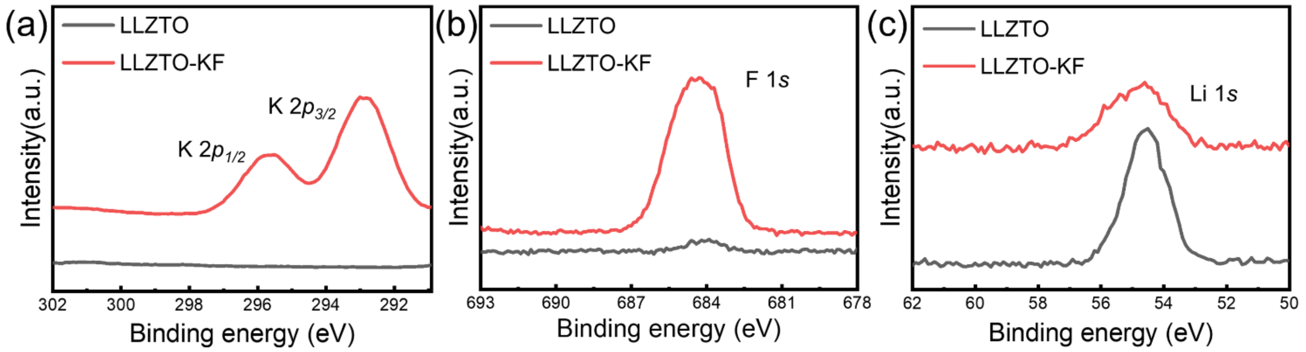

Figure S6. Surface composition of prepared LLZTO pellet and LLZTO-KF pellet. (a-c) XPS spectrum of K 2*p*, F 1*s* and Li 1*s* region of LLZTO and LLZTO-KF.

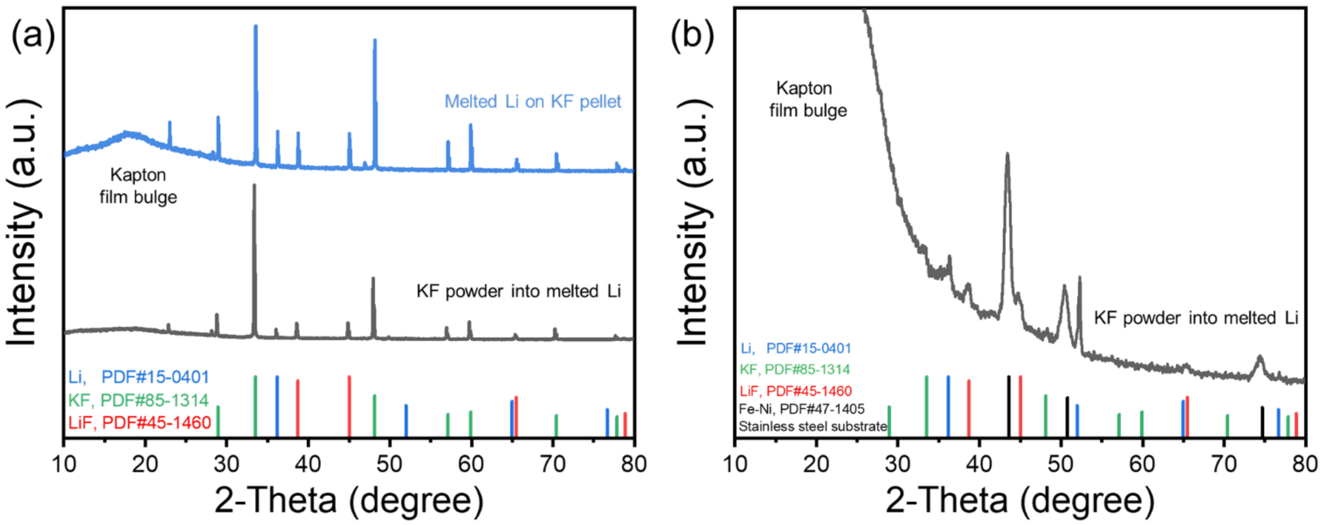

Figure S7. The XRD (a) and GI-XRD (b) patterns with an incidence angle of 1° of Li-KF reaction product under Kapton film.

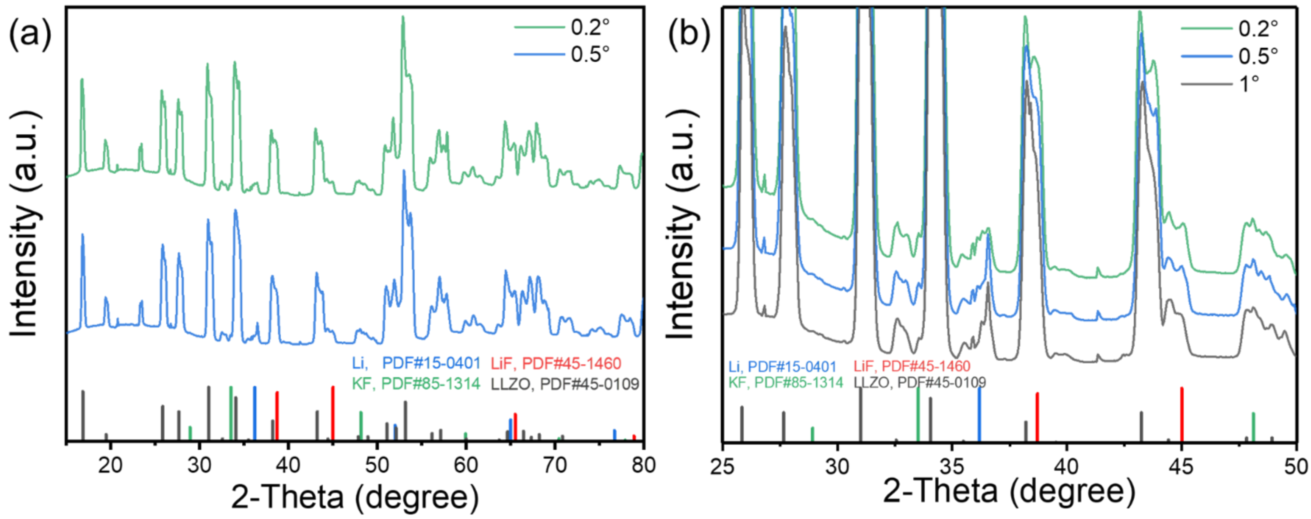

Figure S8. Synchrotron radiation GI-XRD patterns (a) and the enlarged picture (b) of LLZTO-KF|Li sample at the incidence angle of 0.2°, 0.5° and 1°.

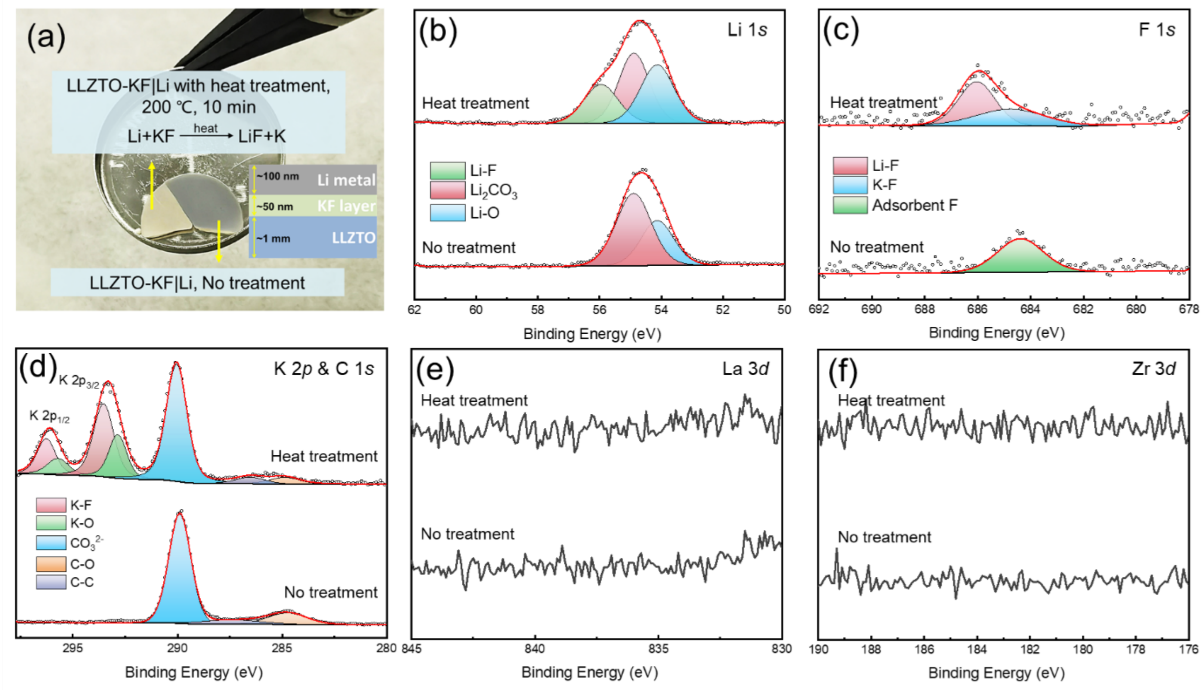

Figure S9. (a) The photos of LLZTO-KF|Li pellet with/without heat treatment. (b-f) XPS spectrum of Li 1*s*, F 1*s*, K 2*p*, La 3*d* and Zr 3*d* regions of LLZTO-KF|Li pellet with/without heat treatment, respectively.

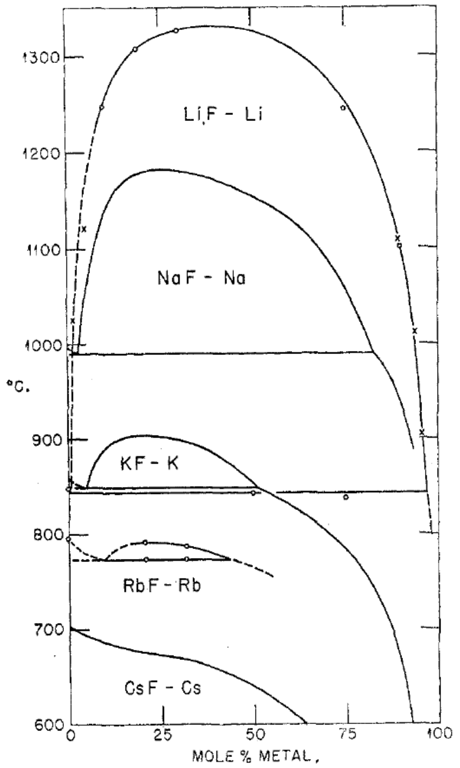

Figure S10. Liquid metal-salt phase equilibria in the alkali metal-fluoride systems (LiF-Li and KF-K systems) [1].

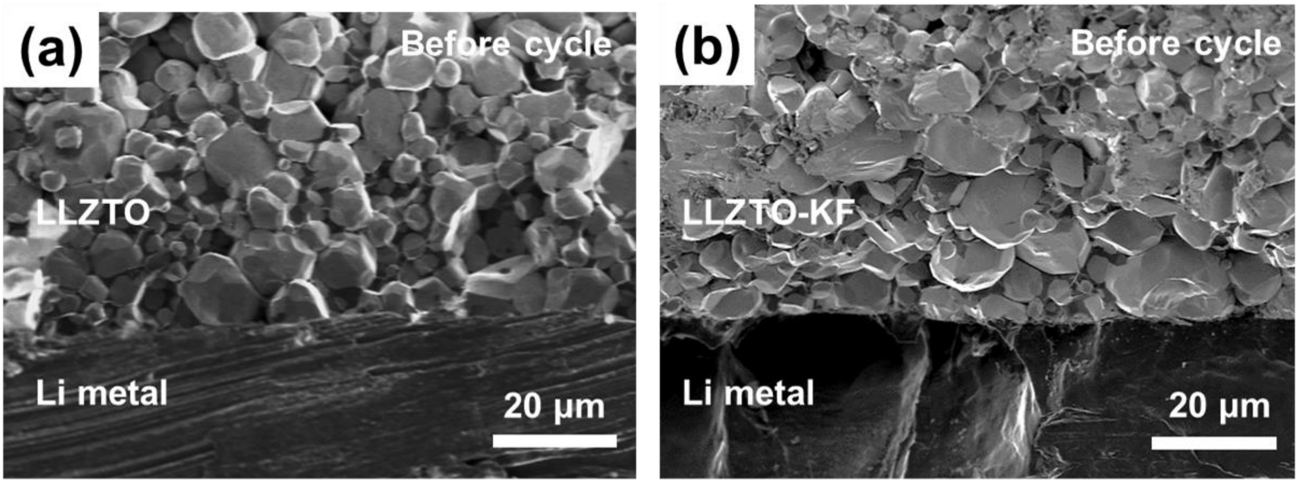

Figure S11. Cross-section SEM images of LLZTO|Li and LLZTO-KF|Li interfaces before cycling.

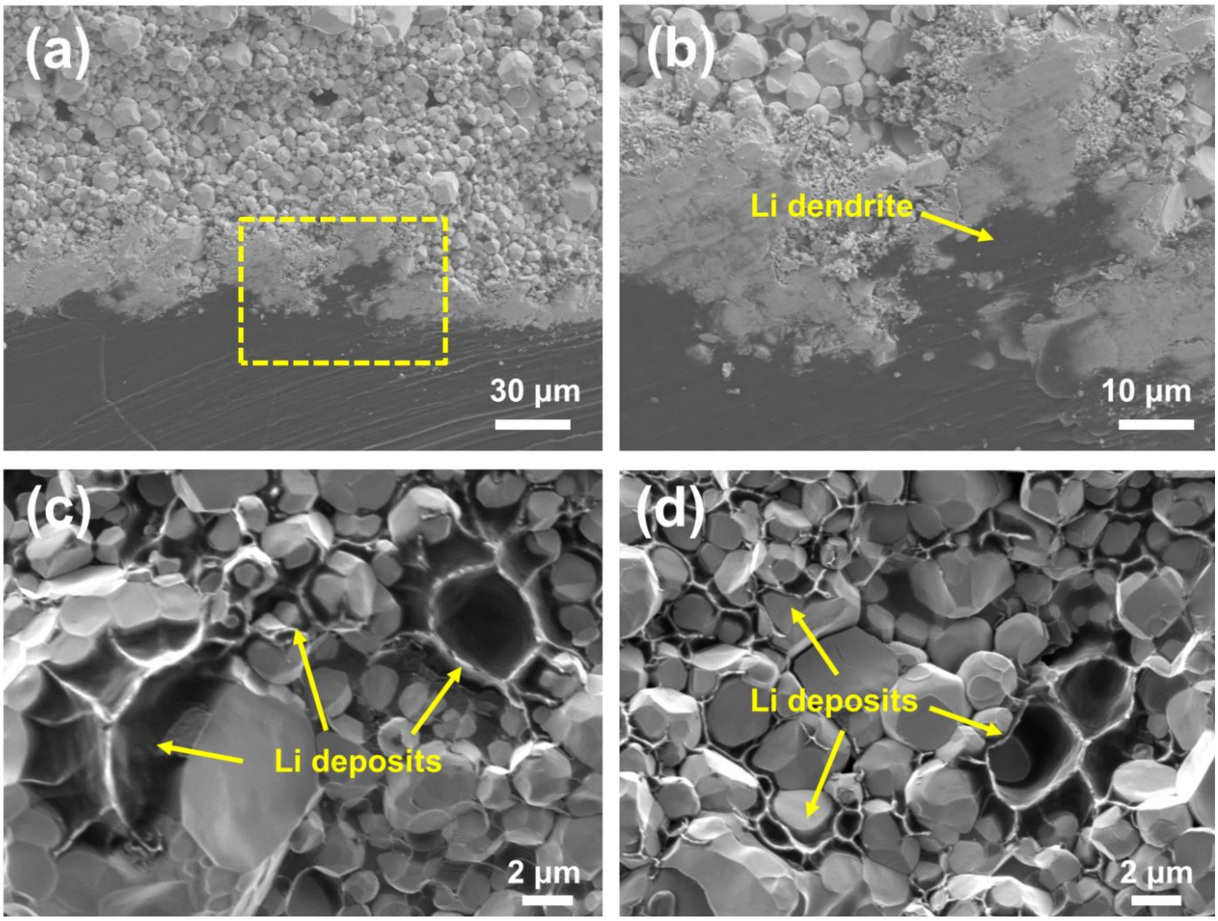

Figure S12. (a, b) Cross-sectional SEM images of LLZTO|Li interface (a) and the enlarged images (b) after cycles. (c, d) SEM images of lithium dendrite in different areas inside LLZTO after cycles.

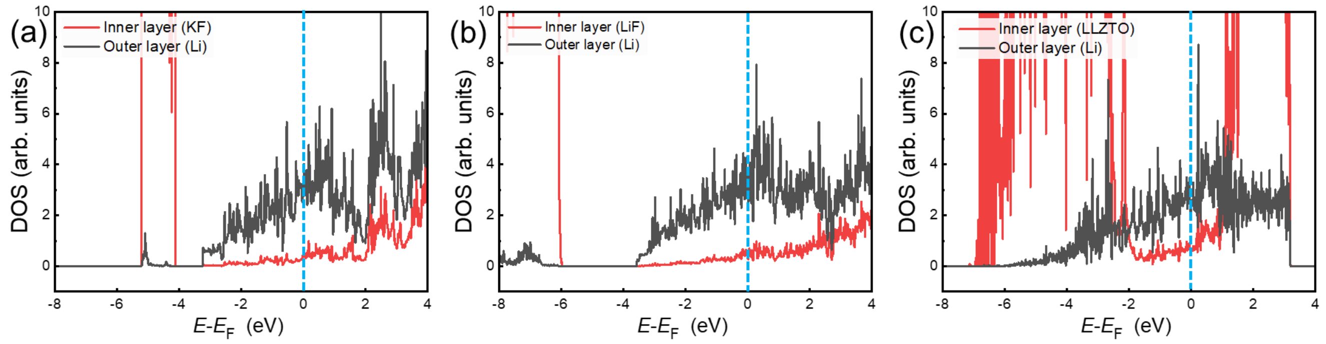

Figure S13. Density of states for the interfaces of KF|Li (a), LiF|Li (b) and LLZTO|Li (c).

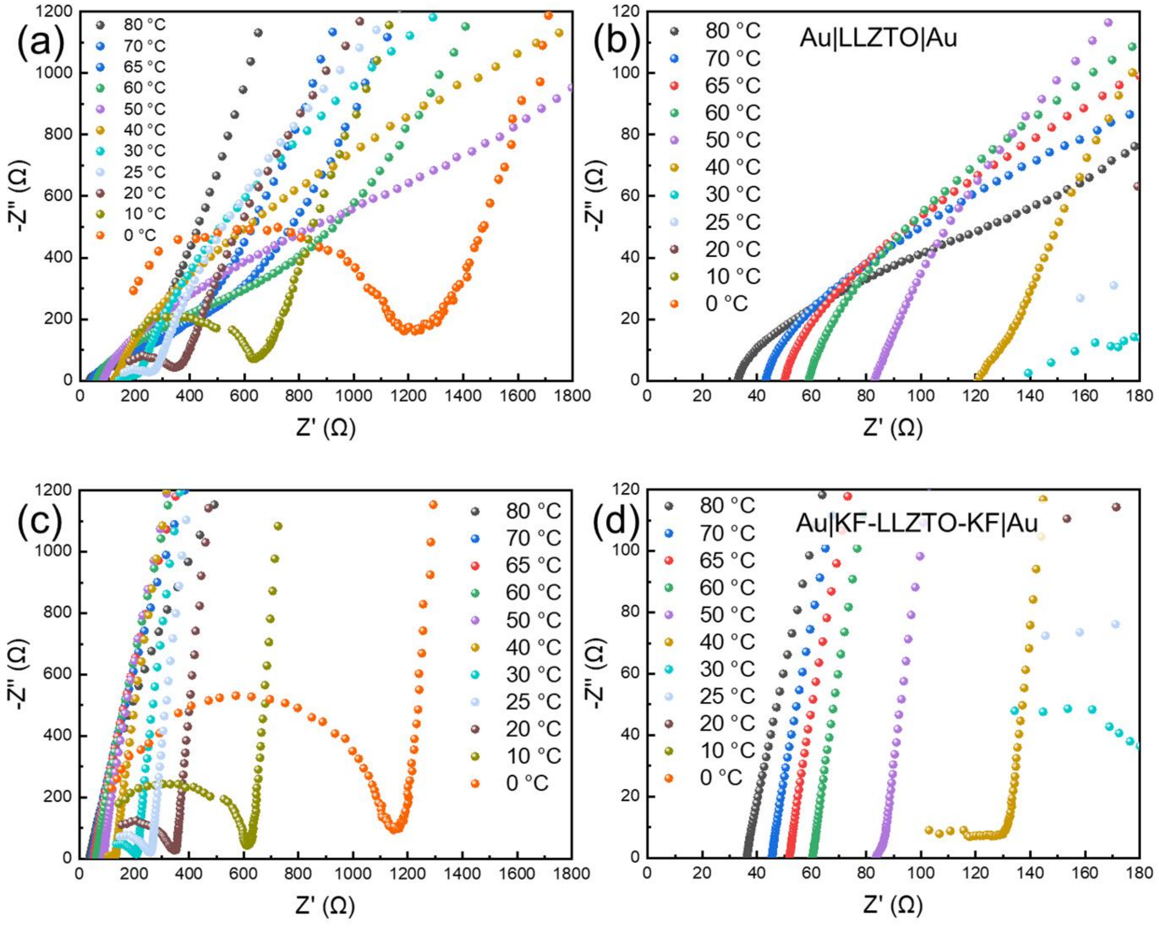

Figure S14. Nyquist plots of Au|LLZTO|Au and Au|KF-LLZTO-KF|Au symmetric cells at various temperatures. (a, b) Nyquist plots and enlarged plots of Au|LLZTO|Au cells. (c, d) Nyquist plots and enlarged plots of Au|KF-LLZTO-KF|Au cells.

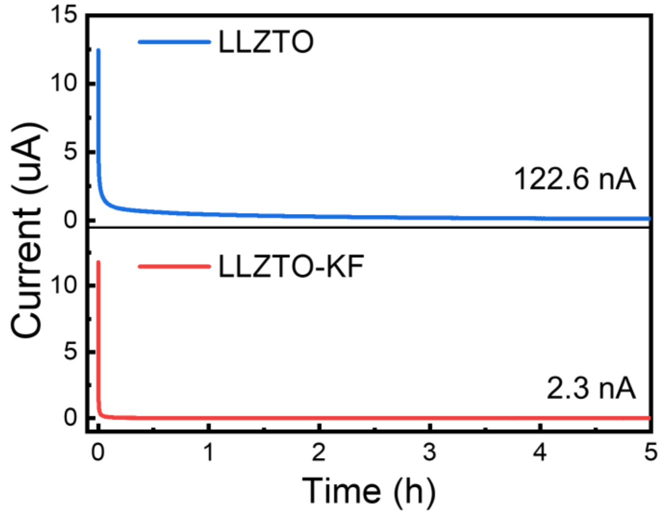

Figure S15. The DC polarization plots of LLZTO and LLZTO-KF solid state electrolyte at 25 °C.

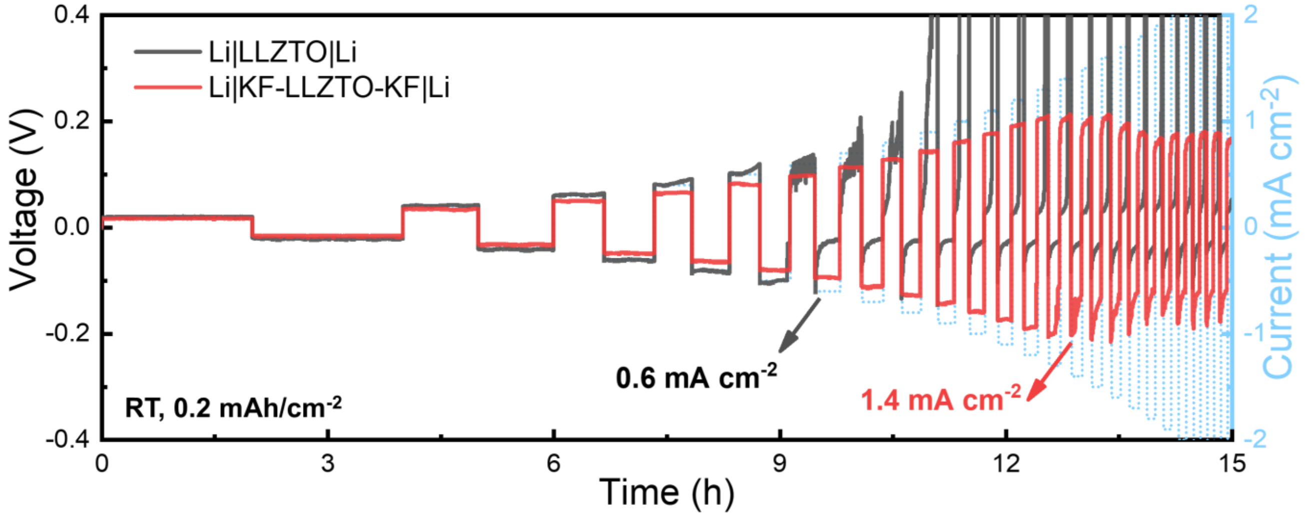

Figure S16. CCD profiles of Li|KF-LLZTO-KF|Li and Li|LLZTO|Li symmetric cells of capacity-constant mode at 25 °C.

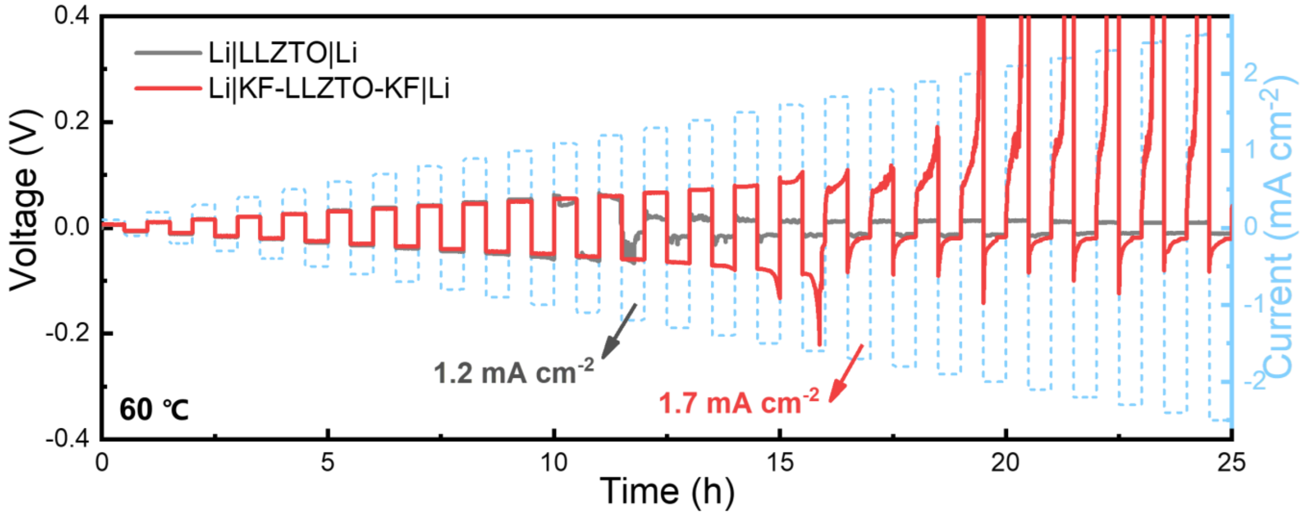

Figure S17. CCD profiles of Li|KF-LLZTO-KF|Li and Li|LLZTO|Li symmetric cells of time-constant mode at 60 °C.

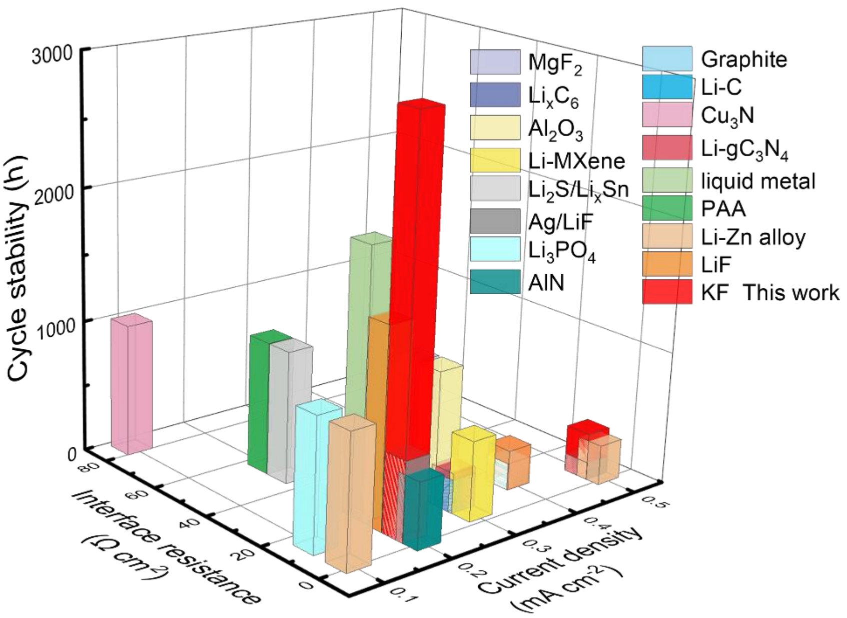

Figure S18. Electrochemical performance comparison chart of our work with other literature reports.

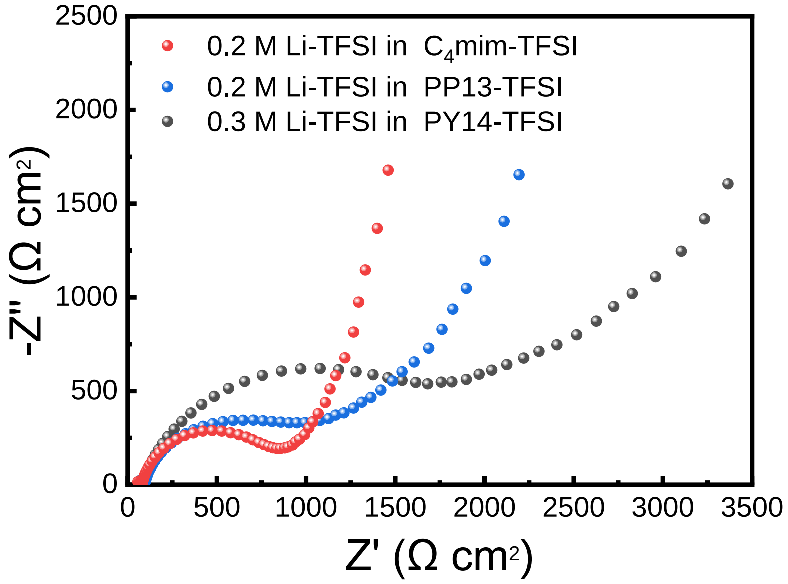

Figure S19. EIS curves of Li|KF-LLZTO-IL|NCM cells with different ionic liquids at 25 °C.

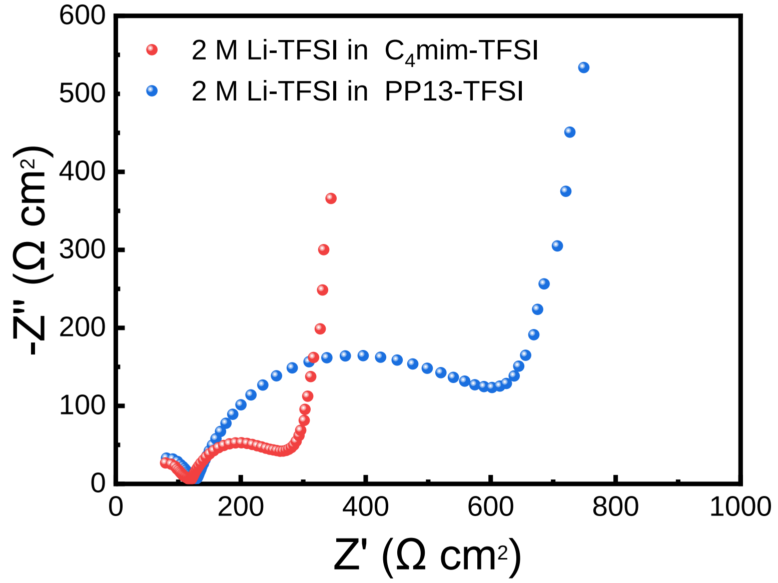

Figure S20. EIS curves of Li|KF-LLZTO-IL|NCM cells with 2 M Li-TFSI in various ionic liquids at 25 °C.

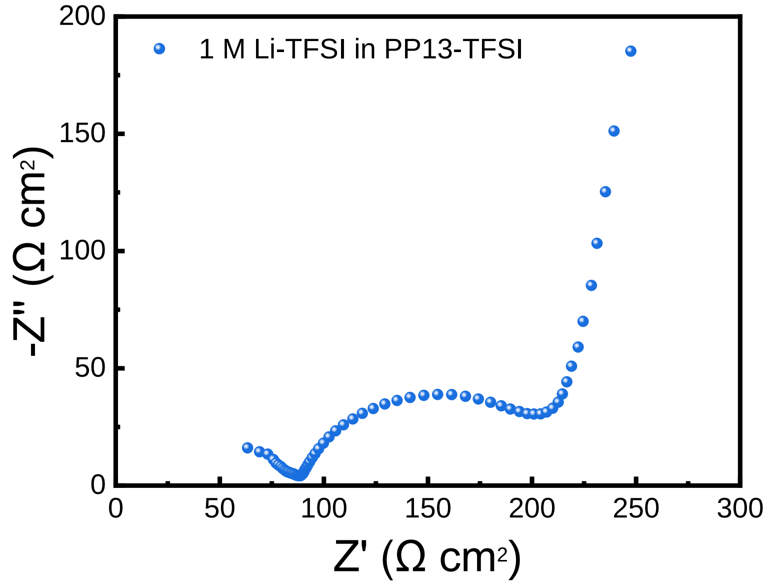

Figure S21. EIS curves of Li|KF-LLZTO-IL|NCM cells with 1 M Li-TFSI in PP13-TFSI ionic liquid at 25 °C.

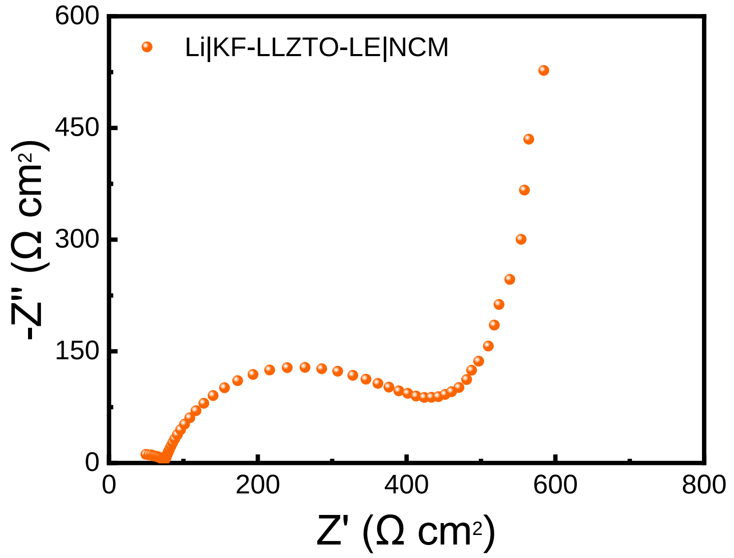

Figure S22. EIS curves of Li|KF-LLZTO-LE|NCM cells at 25 °C. LE is liquid electrolyte, 1 M $LiPF_6$ in EC/DMC (v/v=1:1) with 2%VC.

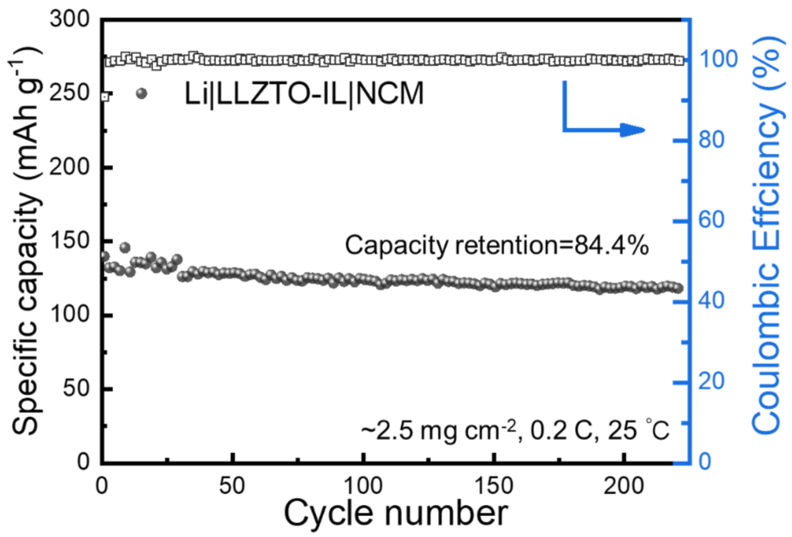

Figure S23. Long cycle performance of Li|LLZTO-IL|NCM cells with mass loading of ~2.5 mg cm^{-2} at 0.2C.

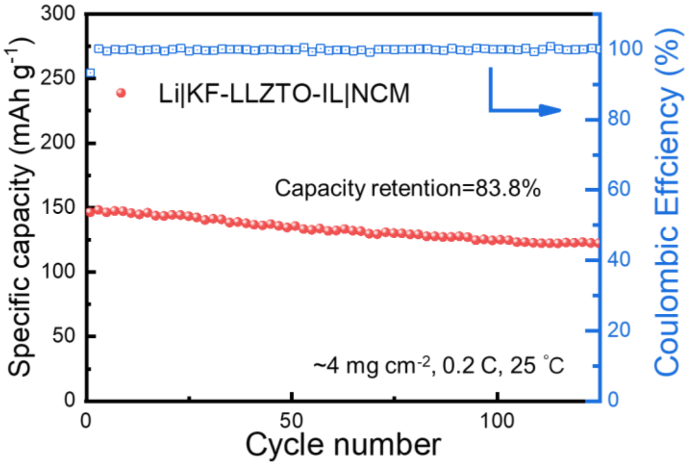

Figure S24. Long cycle performance of Li|KF-LLZTO-IL|NCM cells with mass loading of ~4 mg cm^{-2} at 0.2C.

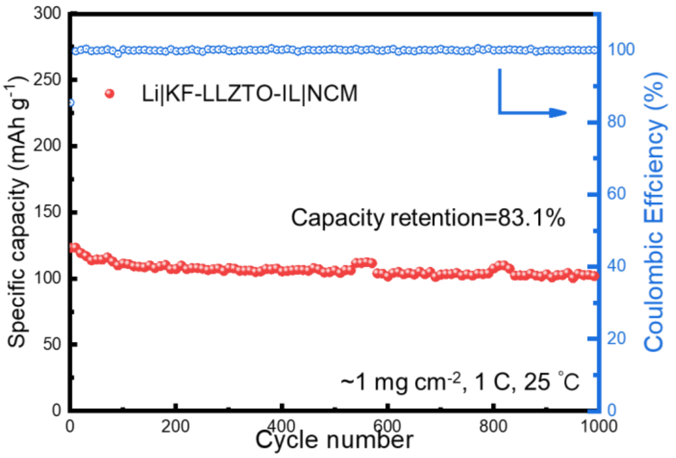

Figure S25. Long cycle performance of Li|KF-LLZTO-IL|NCM cells at 1C.

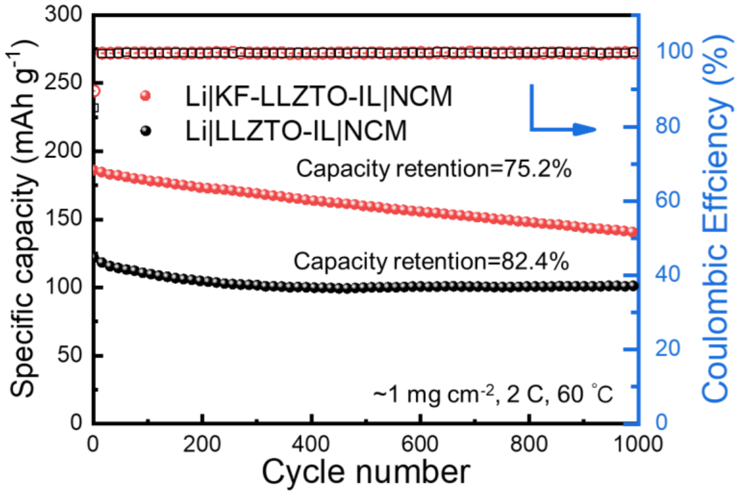

Figure S26. Long cycle performance of Li|KF-LLZTO-IL|NCM cells and Li|LLZTO-IL|NCM cells at 2C and 60 °C.

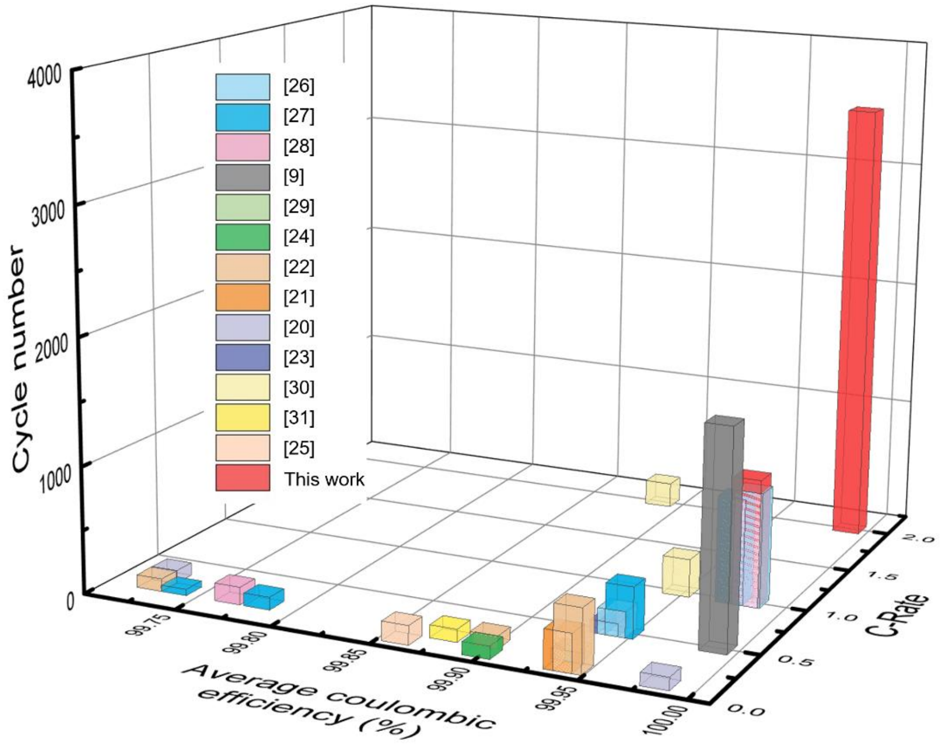

Figure S27. Cycle performance comparison chart of our work with other literature reports.

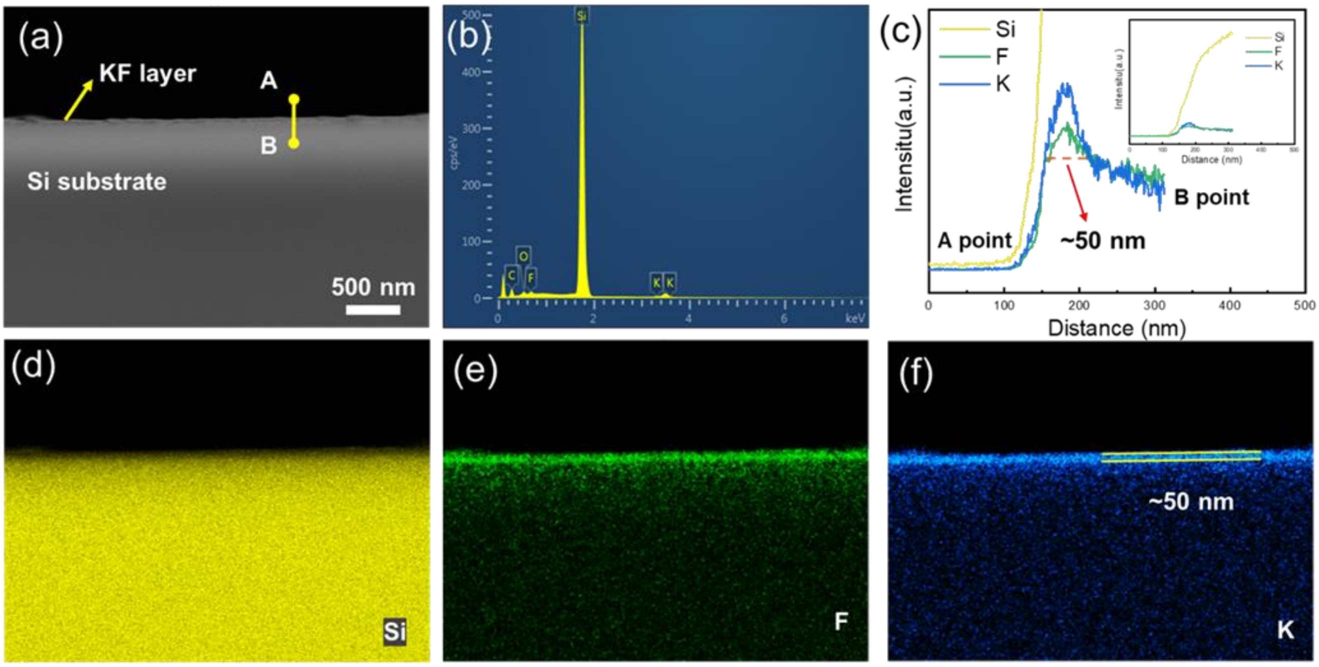

Figure S28. (a) Cross-section SEM image of Si-substrate|KF interface. (b) EDS spectrum of full elements. (c) Line scan EDS spectrum of Si, K and F elements from point A to point B. (d-f) EDS mappings of Si (d), F (e) and K (f) corresponding to the SEM image (a).

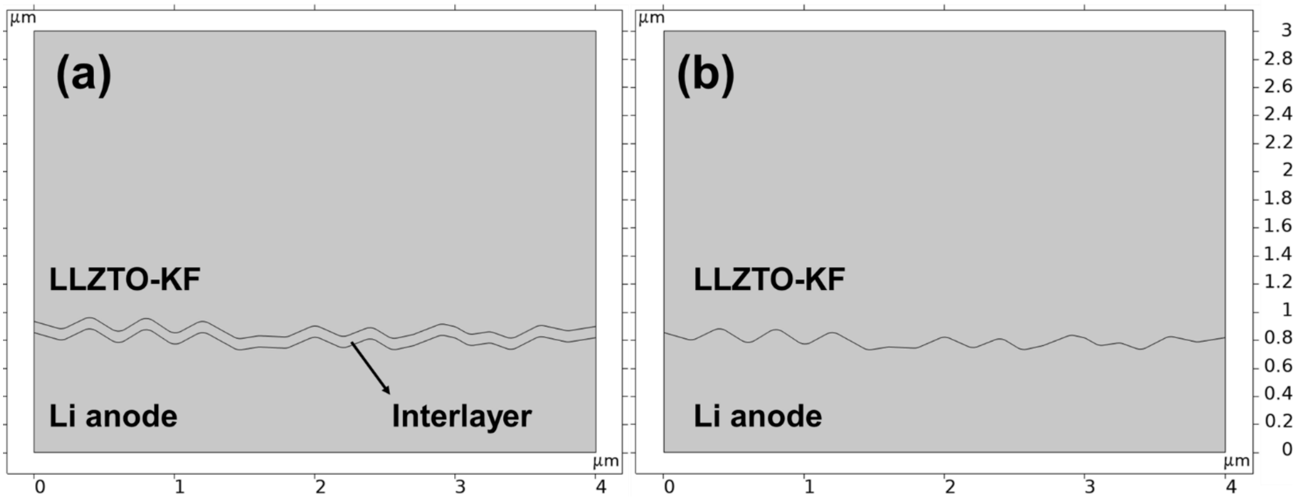

Figure S29. Simulation cell geometry in COMSOL for LLZO-KF|Li interface (a) and LLZO|Li interface (b).

Note S1

Specially, we find more intuitive evidence that the remarkable reaction evolution could be observed directly with the naked eyes. We thermally evaporate ~100 nm lithium metal on LZTO-KF pellet, the surface is covered by gray lithium metal. Then put a piece of LLZTO-KF|Li pellet on hotplate for 200 °C heat treatment for 10 min to accelerate the reaction process. Finally, gray lithium metal on the surface nearly disappeared after heat treatment. Figure S9a exhibited the photo of LLZTO-KF|Li pellet with/without heat treatment. XPS test was performed to analyze the surface chemical constituents. As shown in Figure S9b-d, before heat treatment, the Li_2O and Li_2CO_3 signals were obtained from the thermally evaporated lithium metal surface. Weak adsorbent F 1s peak was collected, but no any KF was found according to XPS spectrum of F 1s and K 2p regions. As the contrast, the gray lithium nearly disappeared after heat treatment. Except as the unreacted Li metal and KF, the generated LiF and K were also observed on the sample surface. No Zr and La signals were collected, indicating that the LLZTO substrate was covered by the generated dense layer completely (Figure S9e-f). This finding proves that heat treatment could promote **Reaction 1.**

$$\text{Li (l)} + \text{KF (s)} \rightarrow \text{LiF (s)} + \text{K (l)} \quad (1)$$

Note S2

We measured the EIS data at various temperatures as shown in figure S13, and draw the Arrhenius plots (ln(σT)~1000/T) as shown in figure 5a. Then we obtained the fitting lines by fitting the data points. For Au|LLZTO|Au and Au|KF-LLZTO-KF|Au symmetric cells, the slopes of fitting lines are -4.636±0.091 and -4.518±0.107, respectively. Therefore, the activation energy (E_a) of LLZTO and LLZTO-KF is calculated as 0.40±0.01 eV and 0.39±0.01 eV, respectively.

Table S1. Electronic conductivity comparation chart of our work with other literature reports.

**Solid state electrolyte**	**Modified methods**	**Electronic conductivity** (S cm^{-1})	**Reference**
Al-doped $Li_7La_3Zr_2O_{12}$	SPS	6.4×10^{-8}	[2]
$Li_7La_{2.75}Ca_{0.25}Zr_{1.65}Nb_{0.25}Co_{0.1}O_{12}$	Doping	6×10^{-8}	[3]
$Li_7La_{2.75}Ca_{0.25}Zr_{1.75}Nb_{0.25}O_{12}$	Doping	3.59×10^{-8}	[4]
$Li_{6.2}La_3(Zr_{0.2}Hf_{0.2}Ti_{0.2}Nb_{0.2}Ta_{0.2})_{0.2}O_{12}$	Doping	2.33×10^{-8}	[5]
Al-doped $Li_7La_3Zr_2O_{12}$	Hot press	2×10^{-8}	[6]
$Li_{6.5}La_3Zr_{1.5}Ta_{0.5}O_{12}$	Rapid sintering	1.2×10^{-8}	[7]
($Li_{6.7}La_3Zr_{1.7}Ta_{0.3}O_{12}$	Doping	5.4×10^{-9}	[8]
$Li_{6.4}La_3Zr_{1.4}Ta_{0.6}O_{12}$	Ag-LiF	5.0×10^{-9}	[9]
$Li_{6.4}La_3Zr_{1.4}Ta_{0.6}O_{12}$	AlN sinter additive	2.6×10^{-9}	[10]
$Li_{6.4}Ga_{0.2}La_3Zr_2O_{12}$	Doping	5×10^{-10}	[11]
$Li_{6.4}La_3Zr_{1.4}Ta_{0.6}O_{12}$	KF layer	1.8×10^{-10}	**This work**

Table S2. Critical current density (CCD), area specific resistance (ASR) and cycling life of the reported Li|LLZO|Li symmetrical cells with different interface layers at 25 °C or other temperatures.

Various LLZO solid state electrolytes	Interface layer	CCD (mA cm^{-2})	ASR (Ω cm^2)	Cycle life (mA cm^{-2}@h)	Year [Ref.]
$Li_{5.9}Al_{0.2}La_3Zr_{1.75}W_{0.25}O_{12}$	Drawing graphite	N/A	25	0.3@1000	2018[12]
$Li_{6.5}La_3Zr_{1.5}Ta_{0.5}O_{12}$	Li-C composite	1.0 (time mode)	11	0.3@250	2019[13]
$Li_{6.4}La_3Zr_{1.4}Ta_{0.6}O_{12}$	Cu_3N film	1.2 (time mode)	83.4	0.1@1000	2020[14]
$Li_{6.5}La_3Zr_{1.5}Ta_{0.5}O_{12}$	Li-gC_3N_4 composite	1.5 (time mode)	11	0.3@300	2020[15]
$Li_{6.5}La_3Zr_{1.5}Ta_{0.5}O_{12}$	liquid metal painting	1.7 (time mode)	19.5	0.2@2000 (25 °C)	2020[16]
$Li_{6.4}La_3Zr_{1.4}Ta_{0.6}O_{12}$	PAA	1.2 (time mode)	54.5	0.2@1000<br>1@400	2021[17]
$Li_{6.4}La_3Zr_{1.4}Ta_{0.6}O_{12}$	3D Li–Zn alloy	2 (time mode)	1.9	0.1@1000<br>0.5@300	2021[18]
$Li_{6.4}La_3Zr_{1.4}Ta_{0.6}O_{12}$	LiF	0.75 (time mode)	12.7	0.2@1500<br>0.4@300	2021[19]
$Li_{6.75}La_3Zr_{1.75}Ta_{0.25}O_{12}$	MgF_2	0.65 (time mode)	25	0.3@1000	2022[20]
$Li_{6.4}La_3Zr_{1.4}Ta_{0.6}O_{12}$	Li_xC_6	N/A	26.2	0.3@900	2022[21]
$Li_{6.5}La_3Zr_{1.5}Ta_{0.5}O_{12}$	Al_2O_3	1.0 (time mode)	16.8	0.3@1000	2022[22]

$Li_{6.75}La_3Za_{1.75}Ta_{0.25}O_{12}$	LiF/Li-Mxene	1.5 (time mode)	5	0.3@600	2022[23]
$Li_{6.75}La_3Za_{1.75}Ta_{0.25}O_{12}$	Li_2S/Li_xSn	0.5 (time mode)	47	0.2@1000	2022[24]
$Li_{6.4}La_3Zr_{1.4}Ta_{0.6}O_{12}$	Ag-LiF	0.75 (time mode)	5.8	0.2@600<br>0.5@130	2022[9]
$Li_{6.4}La_3Zr_{1.4}Ta_{0.6}O_{12}$	Li_3PO_4	0.8 (30 °C) (time mode)	13(30 °C)	0.1@1000 (30 °C)<br>0.4@200 (30 °C)	2022[25]
$Li_{6.4}La_3Zr_{1.4}Ta_{0.6}O_{12}$	AlN	0.76 (time mode)	1	0.2@500	2022[10]
$Li_{6.4}La_3Zr_{1.4}Ta_{0.6}O_{12}$	KF	1.0 (time mode)<br>1.4 (capacity mode)	5.9	0.2@3000<br>0.5@350	**This work**

Table S3. The interface impedance values of Li|KF-LLZTO|NCM cells with various ionic liquids and liquid electrolyte.

	**0.3 M PY14**	**0.2 M PP13**	**0.2 M C_4mim**	**1 M PP13**	**1 M C_4mim**	**2 M PP13**	**2 M C_4mim**	**1 M LE**
**Interface impedance (Ω cm^2)**	1720	1150	710	143	**76**	520	170	340

Table S4. Cycle performance comparation chart of our work with other literature reports.

**Cathode**	**Modifications**	**C-Rate**	**Current density (mA cm^{-2})**	**Mass loading** (mg cm^{-2})	**Cycle numbers**	**Retention (%)**	**Average coulombic efficiency (%)**	**Voltage range (V)**	**Ref.**
$LiFePO_4$	10 μL commercial liquid electrolyte	1C	~0.51	3-4	900	85.4	99.98	2-4	[26]
$LiNi_{0.8}Co_{0.1}Mn_{0.1}O_2$		0.5C	~0.285	3-4	200	89	99.95	3-4.3	
$LiNi_{0.8}Co_{0.1}Mn_{0.1}O_2$	7 μL liquid electrolyte, 1 M $LiPF_6$ in EC/DEC	0.2C	~0.17	~4.5	50	87.7	99.74	2.7-4.3	[27]
$LiFePO_4$		1C	~0.34	~2	835	80.6	99.97	2.5-4	
$LiFePO_4$	7 μL	0.5C	~0.17	~2	400	80.4	99.95	2.5-4	

$LiNi_{0.8}Co_{0.1}Mn_{0.1}O_2$	SN+LiTFSI	0.2C	~0.076	~2	100	80.4	99.78	2.7-4.3	
$LiNi_{0.83}Co_{0.11}Mn_{0.06}O_2$	10 μL ionic liquid	0.2C	0.1	~2	140	72.1	99.77	2.7-4.3	[28]
$LiNi_{1/3}Co_{1/3}Mn_{1/3}O_2$	ionic liquid, 2 M LiFSI in Pyr13FSI	0.5C	0.25	3-3.5	1700	~91	99.99	2.8-4.2	[9]
$LiFePO_4$	10 μL liquid electrolyte, 1 M $LiFP_6$ in EC/DME/EMC	0.2C	0.05	~1.8	80	95	99.94	2.5-4	[29]
$LiFePO_4$	6 μL liquid electrolyte, 1 M $LiFP_6$ in EC/DEC	0.1C	0.068	~4	100	90.4	99.90	2-4.2	[24]
$LiFePO_4$	3 μL ionic liquid, 0.3M LiTFSI in PY14TFSI	0.1C	N/A	N/A	500	~75	99.94	2.5-4	[22]
$LiNi_{0.83}Co_{0.11}Mn_{0.06}O_2$		0.2C	~0.192	~4.8	100	90.2	99.90	3-4.3	
$LiNi_{0.83}Co_{0.11}Mn_{0.06}O_2$	liquid electrolyte	0.2C	~0.192	~4.8	100	75.9	99.72	3-4.3	
$LiFePO_4$	3 μL liquid electrolyte, 1 M $LiFP_6$ in EC/DME/EMC	0.1C	~0.029	~1.8	300	83	99.94	2.5-4.2	[21]
$LiFePO_4$	6 μL SN+LiTFSI	0.3C	~0.2	~4	100	75.9	99.72	2.5-4.2	[20]
$LiFePO_4$		0.1C	~0.068	~4	100	98.3	99.98	2.5-4.2	
$LiFePO_4$	10 μL liquid electrolyte, 1 M $LiPF_6$ in EC/DEC	0.5C	~0.208	2.45	100	94	99.94	2.5-4.2	[23]
$LiNi_{0.5}Co_{0.3}Mn_{0.2}O_2$	15 μL liquid electrolyte	1C	~0.65	~3.8	300	85.5	99.95	2.8-4.3	[30]
$LiNi_{0.5}Co_{0.3}Mn_{0.2}O_2$		2C	~1.3	~3.8	200	80%	97.61	2.8-4.3	
$LiNi_{0.6}Co_{0.2}Mn_{0.2}O_2$	10 μL liquid electrolyte, 1 M $LiPF_6$ in EC/DMC	0.2C			100	88.43	99.88	2.8-4.5	[31]

$LiFePO_4$	3 μL ionic liquid, 0.2 M LiTFSI in PP13-TFSI	0.1C (30 °C)			150	81	99.86	3-4.2	[25]
$LiNi_{0.8}Co_{0.1}Mn_{0.1}O_2$	3 μL ionic liquid, 1 M LiTFSI in C_4mim-TFSI	0.2C	~0.095	~2.5	270	82.8	99.93	2.8-4.3	**This work**
$LiNi_{0.8}Co_{0.1}Mn_{0.1}O_2$		1C	~0.475	~2.5	1000	83.1	99.98	2.8-4.3	
$LiNi_{0.8}Co_{0.1}Mn_{0.1}O_2$		2C	~0.38	~1.0	3000	72.1	99.99	2.8-4.3	

Table S5. *The COMSOL simulation details.*

Parameter	Value
Geometrical size	4×3 μm^2
Electrolyte conductivity	5×10^{-4} S cm^{-1}
Electrode conductivity	1×10^{7} S cm^{-1}
Applied current density	0.1 mA cm^{-2}
Reaction electron number	1
Reference exchange current density (LLZTO)	100 A m^2
Reference exchange current density (LLZTO-KF)	1000 A m^2

Table S6. The DFT calculation details for LLZTO/Li, KF/Li, and LiF/Li.

Parameters	Settings		
Pseudopotential type	Projector augmented wave (PAW)		
Exchange-correlation functional	Generalized gradient approximation (GGA) of Perdew–Burke–Ernzerh (PBE)		
Valence electron configurations	Li-$2s^1$, O-$2s^2 2p^4$, F-$2s^2 2p^5$, K-$4s^1$, Zr-$5s^2 4d^2$, La-$5d^1 6s^2$, Ta-$5d^3 6s^2$		
Precision mode	Accurate		
Cutoff energy (eV)	400		
Electronic convergence (eV)	1×10^{-4}		
Systems	LLZTO/Li	KF/Li	LiF/Li
Number of atoms	152 Li<br>24 La<br>12 Zr<br>4 Ta<br>96 O<br>288 in total	72 K<br>72 F<br>96 Li<br>240 in total	72 F<br>168 Li<br>240 in total
k-point meshes for relaxation	2×2×1	2×2×1	2×2×1
k-point meshes for DOS calculation	4×4×2	6×6×3	6×6×3
Center of the meshes	Gamma centered mesh.		

Reference

[1] A. Dworkin;H. Bronstein;M. J. T. J. o. P. C. Bredig, **1962** *66*, 572-573.

[2] Y. Zhu, J. Zhang, W. Li, Y. Zeng, W. Wang, Z. Yin, B. Hao, Q. Meng, Y. Xue, J. Yang, and S. Li, *Journal of Alloys and Compounds* **2023** *967*.

[3] A. J. Samson;K. Hofstetter;E. Wachsman;V. Thangadurai, *Journal of the Electrochemical Society* **2018** *165*, A2303-A2311.

[4] Y. L. Song;L. Y. Yang;W. G. Zhao;Z. J. Wang;Y. Zhao;Z. Q. Wang;Q. H. Zhao;H. Liu;F. Pan, *Advanced Energy Materials* **2019** *9*.

[5] S. X. Han;Z. Q. Wang;Y. Ma;Y. Miao;X. M. Wang;Y. Wang;Y. Z. Wang, *Journal of Advanced Ceramics* **2023** *12*, 1201-1213.

[6] E. Rangasamy;J. Wolfenstine;J. Sakamoto, *Solid State Ionics* **2012** *206*, 28-32.

[7] L. Yang;Q. S. Dai;L. Liu;D. S. Shao;K. L. Luo;S. Jamil;H. Liu;Z. G. Luo;B. B. Chang;X. Y. Wang, *Ceramics International* **2020** *46*, 10917-10924.

[8] M. Y. Yi;T. Liu;X. N. Wang;J. Y. Li;C. Wang;Y. C. Mo, *Ceramics International* **2019** *45*, 786-792.

[9] S. Lee;K. S. Lee;S. Kim;K. Yoon;S. Han;M. H. Lee;Y. Ko;J. H. Noh;W. Kim;K. Kang, *Science Advances* **2022** *8*.

[10] C. Zhang, X. C. Hu, Z. W. Nie, C. Wu, N. Zheng, S. J. Chen, Y. H. Yang, R. Wei, J. M. Yu, N. Yang, Y. Yu, and W. Liu, *Journal of Advanced Ceramics* **2022** *11*, 1530-1541.

[11] M. Philipp;B. Gadermaier;P. Posch;I. Hanzu;S. Ganschow;M. Meven;D. Rettenwander;G. J. Redhammer;H. M. R. Wilkening, *Advanced Materials Interfaces* **2020** *7*.

[12] Y. J. Shao, H. C. Wang, Z. L. Gong, D. W. Wang, B. Z. Zheng, J. P. Zhu, Y. X. Lu, Y. S. Hu, X. X. Guo, H. Li, X. J. Huang, Y. Yang, C. W. Nan, and L. Q. Chen, *Acs Energy Letters* **2018** *3*, 1212-1218.

[13] J. Duan;W. Y. Wu;A. M. Nolan;T. R. Wang;J. Y. Wen;C. C. Hu;Y. F. Mo;W. Luo;Y. H. Huang, *Advanced Materials* **2019** *31*.

[14] H. Y. Huo;Y. Chen;R. Y. Li;N. Zhao;J. Luo;J. G. P. da Silva;R. Mucke;P. Kaghazchi;X. X. Guo;X. L. Sun, *Energy & Environmental Science* **2020** *13*, 127-134.

[15] Y. Huang;B. Chen;J. Duan;F. Yang;T. R. Wang;Z. F. Wang;W. J. Yang;C. C. Hu;W. Luo;Y. H. Huang, *Angewandte Chemie-International Edition* **2020** *59*, 3699-3704.

[16] J. W. Meng;Y. Zhang;X. J. Zhou;M. Lei;C. L. Li, *Nature Communications* **2020** *11*.

[17] H. Y. Huo, J. Gao, N. Zhao, D. X. Zhang, N. G. Holmes, X. N. Li, Y. P. Sun, J. M. Fu, R. Y. Li, X. X. Guo, and X. L. Sun, *Nature Communications* **2021** *12*.

[18] Z. P. Wan;K. Shi;Y. F. Huang;L. Yang;Q. B. Yun;L. K. Chen;F. Z. Ren;F. Y. Kang;Y. B. He, *Journal of Power Sources* **2021** *505*.

[19] S. J. Tang;G. W. Chen;F. C. Ren;H. C. Wang;W. Yang;C. X. Zheng;Z. L. Gong;Y. Yang, *Journal of Materials Chemistry A* **2021** *9*, 3576-3583.

[20] J. L. Jiang;Y. H. Ou;S. Y. Lu;C. Shen;B. B. Li;X. Y. Liu;Y. Jiang;B. Zhao;J. J. Zhang, *Energy Storage Materials* **2022** *50*, 810-818.

[21] C. Cui;Q. Ye;C. Zeng;S. H. Wang;X. W. Xu;T. Y. Zhai;H. Q. Li, *Energy Storage Materials* **2022** *45*, 814-820.

[22] S. J. Guo, Y. T. Li, B. Li, N. S. Grundish, A. M. Cao, Y. G. Sun, Y. S. Xu, Y. L. M. Ji, Y. Qiao, Q. H. Zhang, F. Q. Meng, Z. H. Zhao, D. Wang, X. Zhang, L. Gu, X. Q. Yu, and L. J. Wan, *Journal of the American Chemical Society* **2022** *144*, 2179-2188.

[23] J. Y. Wen;L. Q. Huang;Y. Huang;W. Luo;H. Y. Huo;Z. F. Wang;X. Y. Zheng;Z. Y. Wen;Y. H. Huang, *Energy Storage Materials* **2022** *45*, 934-940.

[24] B. Zhao;W. C. Ma;B. B. Li;X. T. Hu;S. Y. Lu;X. Y. Liu;Y. Jiang;J. J. Zhang, *Nano Energy* **2022** *91*.

[25] Z. Bi;Q. Sun;M. Jia;M. Zuo;N. Zhao;X. Guo, *Advanced Functional Materials* **2022** *32*.

[26] B. Chen;J. Zhang;T. Zhang;R. Wang;J. Zheng;Y. Zhai;X. Liu, *Advanced Science* **2023** *10*, 2207056.

[27] G. J. Lu;W. Liu;Z. G. Yang;Y. M. Wang;W. K. Zheng;R. R. Deng;R. H. Wang;L. Lu;C. H. Xu, *Advanced Functional Materials* **2023**.

[28] W. J. Ji, B. Luo, Q. Wang, G. H. Yu, Z. H. Liu, Z. W. Zhao, R. R. Zhao, S. B. Wang, X. W. Wang, B. Zhang, J. F. Zhang, F. Hou, and J. Liang, *Advanced Energy Materials* **2023** *13*.

[29] J. X. Zhang;C. H. Wang;M. T. Zheng;M. H. Ye;H. Y. Zhai;J. Li;G. J. Tan;X. F. Tang;X. L. Sun, *Nano Energy* **2022** *102*.

[30] T. R. Wang, J. Duan, B. Zhang, W. Luo, X. Ji, H. H. Xu, Y. Huang, L. Q. Huang, Z. Y. Song, J. Y. Wen, C. S. Wang, Y. H. Huang, and J. B. Goodenough, *Energy & Environmental Science* **2022** *15*, 1325-1333.

[31] C. J. Zheng;J. M. Su;Z. Song;T. P. Xiu;J. Jin;M. E. Badding;Z. Y. Wen, *Materials Today Energy* **2022** *27*.
